# Dynamic control of coherent pulses via destructive interference in graphene under Landau quantization

**DOI:** 10.1038/s41598-017-02740-x

**Published:** 2017-05-31

**Authors:** Wen-Xing Yang, Ai-Xi Chen, Xiao-Tao Xie, Shaopeng Liu, Shasha Liu

**Affiliations:** 1 Department of Physics, Southeast University, Nanjing, 211189 China; 2 Department of Physics, Zhejiang Sci-Tech University, Hangzhou, 310018 China; 3 Institute for Quantum Computing, University of Waterloo, Ontario, N2L 3G1 Canada; 4 School of Physics and Information Technology, Shanxi Normal University, Xi’an, 710062 China

## Abstract

We analyze the destructive interference in monolayer graphene under Landau quantization in a time-dependent way by using the Bloch-Maxwell formalism. Based on this analysis, we investigate the dynamics control of an infrared probe and a terahertz (THz) switch pulses in graphene. In presence of the THz switch pulse, the destructive interference take places and can be optimized so that the monolayer graphene is completely transparent to the infrared probe pulse. In absence of the THz switch pulse, however, the infrared probe pulse is absorbed due to such a interference does not take place. Furthermore, we provide a clear physics insight of this destructive interference by using the classical dressed-state theory. Conversely, the present model may be rendered either absorbing or transparent to the THz switch pulse. By choosing appropriate wave form of the probe and switch pulses, we show that both infrared probe and THz switch pulses exhibit the steplike transitions between absorption and transparency. Such steplike transitions can be used to devise a versatile quantum interference-based solid-state optical switching with distinct wave-lengths for optical communication devices.

## Introduction

The discovery of graphene opened a new area in material science. Graphene is the first truly two-dimensional (2D) crystal consisting of just a single layer of carbon atoms arranged in a hexagonal lattice^1^. The graphene has led to many significant research activities on its fascinating electronic and optical properties due to linear and massless band structure near the Dirac point and chiral character of electron states. The magneto-optical properties and thin graphite layers have triggered multiple absorption peaks and particular selection rules between Landau levels (LLs)^2, 3^. The influence of the Landau quantization on the carrier dynamics in graphene has been investigated^4–8^. Progress in making high-quality epitaxial graphene and graphite with high room-temperature mobility and strong magneto-optical response attracted a lot of interest and showed the promise of new applications in the infrared optics and photonics^9–12^. In particular, optical transition between LLs may construct the quantum interference pathways which can be exploited, e.g., to devise a novel-type all-optical switching mechanism.

On the other hand, quantum optical phenomena where the dynamics of one probe pulse can be modulated in time by another pulse have been the focus of current investigations in quantum optics^13–15^. Although a lots of quantum optical effects have been observed in the past decades and within different physics systems, there is renewed interest in such time-dependent dynamic effects which may include solitons^16–20^, creation of short pulses^21–25^, and multiwave mixing processes in the ultraslow propagation regime^26–28^, etc.^29–31^. In particular, time-dependent coherent control of the optical response of certain media that exhibit electromagnetic induced transparency (EIT)^13–15^ has also triggered a flurry of interest mainly due to its potential applications in integrated optics and quantum information science. The LLs transitions are particular attractive for these investigations, which is much more practical than that in atomic system because of its flexible design and the controllable LLs energies.

In this paper, we investigate the time-dependent coherent control of an infrared probe pulse and a terahertz (THz) pulse in a quantized three-level graphene system that exhibit destructive interference between three LLs transitions. This is a rather relevant issue which has remained so far unexplored, as far as we know, both theoretically and experimentally. A few authors have discussed the topic of coherent control of the LLs transitions in graphene system under a strong magnetic field^2–12^, but they mainly focused on the absorption spectra, enhanced nonlinear parametric generation and relaxation dynamics while no time-dependent dynamics of coherent pulses was considered. In ref. 32, pulse propagation dynamics was considered and examined for a quantized four-level graphene system under a mechanism of four-wave mixing by utilizing density-matrix method and perturbation theory, which again is different from our quantized three-level graphene model under a mechanism of destructive interference. The present work also differs from the steady-state treatment of similar theoretical models studied, e.g., in refs 3, 10, 12, and which inherently miss any time-dependent dynamics.

Specifically, we analyze the destructive interference in monolayer graphene under Landau quantization in a time-dependent way via the Bloch-Maxwell formalism. Based on this analysis, we investigate the dynamics control of an infrared probe and a THz switch pulses in graphene. By solving the Bloch-Maxwell equation, we find that the graphene system is completely transparent to the infrared probe pulse when such an interference induced by the switch pulse take places. However, when the THz switch pulse is switched off, the infrared probe pulse is absorbed. Interestingly enough, the present graphene system may also be rendered either absorbing or transparent to the THz switch pulse. Furthermore, we show that both infrared probe and THz switch pulses exhibit the steplike transitions between absorption and transparency. Such steplike transitions can be used to devise a versatile optical switching based on destructive interference for optical communication devices.

## The theoretical model and basic equations

**Figure 1:**
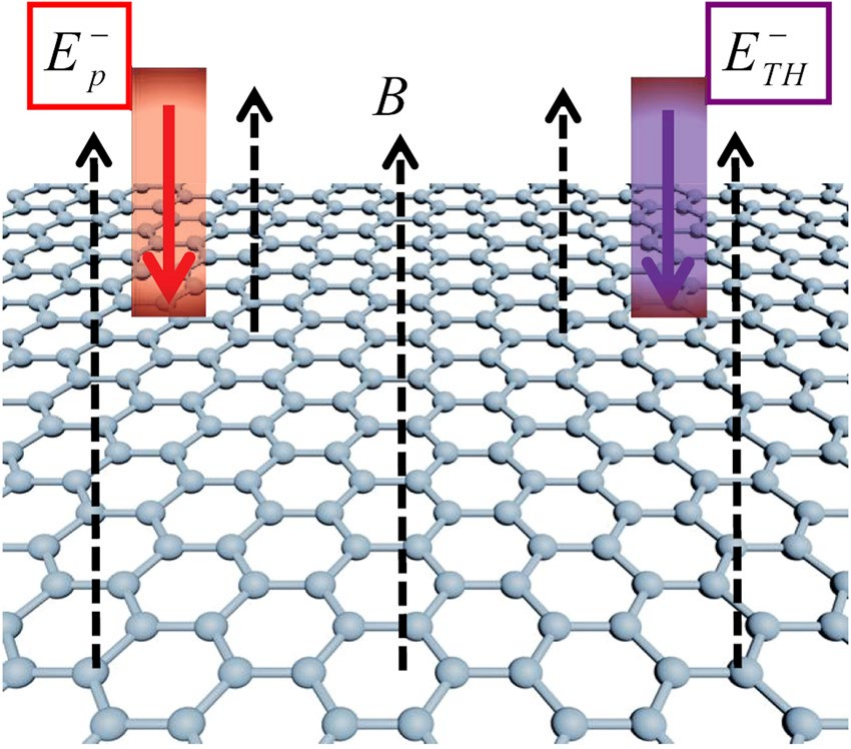
The geometry of the proposed scheme. The infrared probe pulse and THz switch pulse are perpendicularly incident on the single-layer graphene (the monolayer graphene is regarded as a perfect two-dimensional (2D) crystal structure in the *x* − *y* plane) placed in a magnetic field *B*, in which both two optical fields and magnetic field are along the *z*-axis.

We consider a 2D graphene crystal structure in the presence of a strong magnetic field. The geometry of the proposed scheme is shown in Fig. 1. The infrared probe pulse with frequency *ω*_*p*_ and THz switch pulse with frequency *ω*_*TH*_ are perpendicularly incident on the single-layer graphene (the monolayer graphene is regarded as a perfect two-dimensional (2D) crystal structure in the *x* − *y* plane) placed in a magnetic field *B*, in which both two optical fields and magnetic field are along the *z*-axis. Under the action of the external magnetic field, the original linear dispersion relation of graphene results in unequally spaced Landau levels (LLs), their transition energies are proportional to $\sqrt{B}$ ^9–12^.

**Figure 2:**
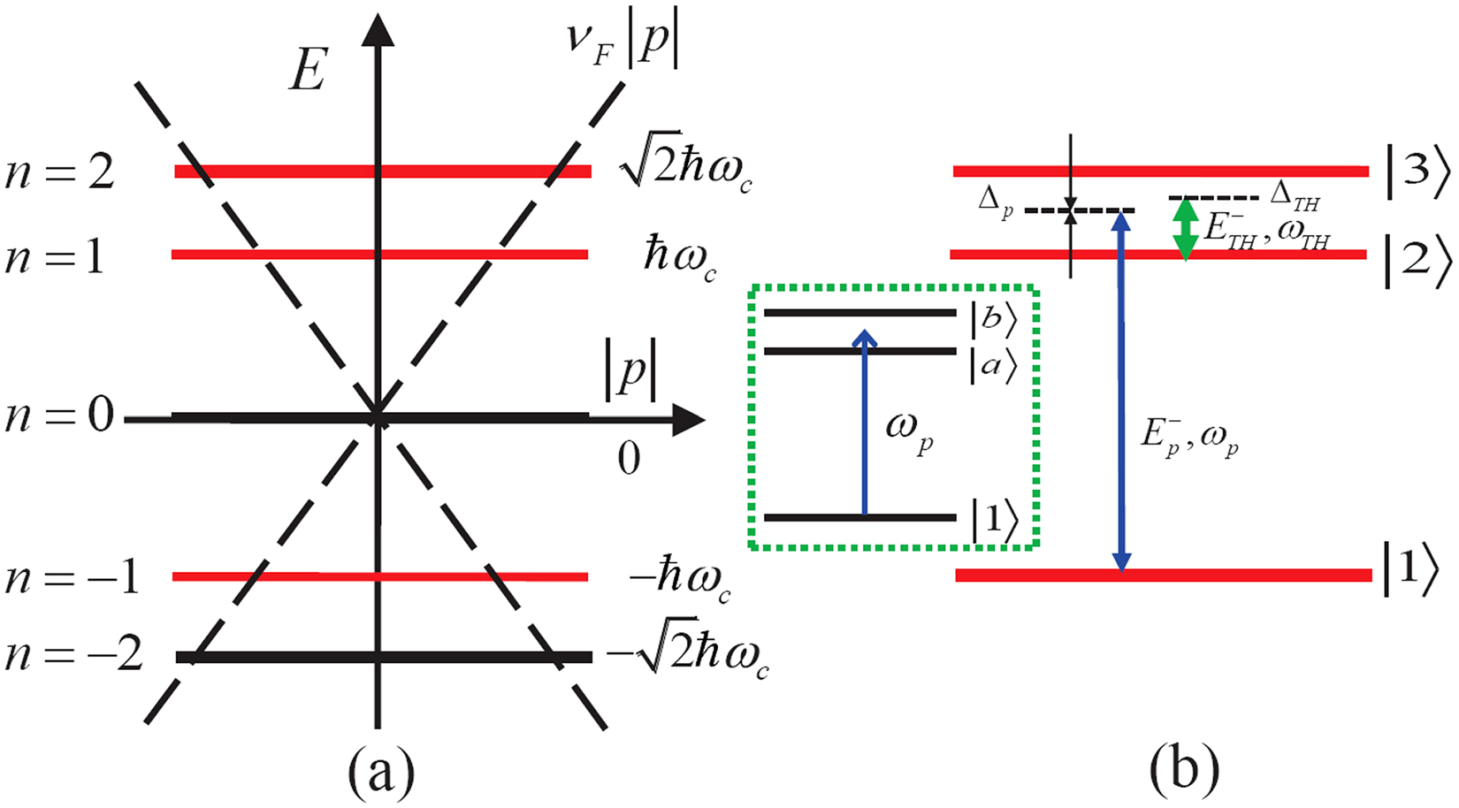
(**a**) Landau levels (LLs) near the Dirac point superimposed on the linear electron dispersion without the magnetic field $E=\pm {\upsilon }_{F}|p|$. The magnetic field condenses the original states in the Dirac cone into discrete energies. (**b**) Energy level diagram and optical transitions in graphene interacting with a weak probe pulse (with carrier frequency *ω*_*p*_) and a THz switch pulse (with carrier frequency *ω*_*TH*_). The states are labeled as |1〉, |2〉 and |3〉 corresponding to the LLs with energy quantum numbers *n* = −1, 1, 2, respectively. The monolayer graphene is regarded as a perfect two-dimensional (2D) crystal structure in the *x* − *y* plane. *Inset*: in the presence of the switch pulse, states |2〉 and |3〉 couple with the switch pulse giving rise to the three dressed states |*a*〉 and |*b*〉.

When the magnetic field is perpendicularly applied in a single-layer graphene (in the *x* − *y* plane), the effective-mass Hamiltonian^9–12^ without external optical field can be written as

1 $${\hat{H}}_{0}={\upsilon }_{F}\,(\begin{array}{cccc}0 & {\hat{\pi }}_{x}-i{\hat{\pi }}_{y} & 0 & 0\\ {\hat{\pi }}_{x}+i{\hat{\pi }}_{y} & 0 & 0 & 0\\ 0 & 0 & 0 & {\hat{\pi }}_{x}+i{\hat{\pi }}_{y}\\ 0 & 0 & {\hat{\pi }}_{x}-i{\hat{\pi }}_{y} & 0\end{array}),$$

where Fermi velocity ${\upsilon }_{F}=3{\gamma }_{0}/2\hslash a\approx {10}^{6}\,m/s$ is a band parameter with the nearest-neighbor hopping energy *γ*_0_ ~ 2.8 eV and C-C spacing *a* = 1.42 *Å*, $\hat{\overrightarrow{\pi }}=\hat{\overrightarrow{p}}+e\overrightarrow{A}/c$ represents the generalized momentum operator, $\hat{\overrightarrow{p}}$ is the electron momentum operator, *e* is the electron charge and $\overrightarrow{A}$ is the vector potential, which is equal to (0, *Bx*) for a uniform magnetic field. In general, one can obtain the eigenenergies of discrete LLs for the magnetized graphene by solving the effective mass Schrödinger equations, i.e., ${\hat{H}}_{0}{\rm{\Psi }}=\varepsilon {\rm{\Psi }}$. In fact, the Hamiltonian near the K point can be expressed as ${\hat{H}}_{0}={\upsilon }_{F}\hat{\overrightarrow{\sigma }}\cdot \hat{\overrightarrow{\pi }}$, where $\hat{\overrightarrow{\sigma }}=({\hat{\sigma }}_{x},{\hat{\sigma }}_{y})$ is a vector of Pauli matrices. Then, the eigenfunction is specified by two quantum numbers *n* ($n=0,\pm 1,\pm 2,\ldots$) and the electron wavevector *k*_*y*_ along *y* direction^9–12^:

2 $${{\rm{\Psi }}}_{n,{k}_{y}}(r)=\frac{{C}_{n}}{\sqrt{L}}\,{{\rm{e}}}^{(-i{k}_{y}y)}(\begin{array}{c}{\rm{sgn}}\,(n)\,{i}^{|n|-1}{\phi }_{|n|-1}\\ {i}^{|n|}{\phi }_{|n|}\end{array})$$

with

3 $${C}_{n}=\{\begin{array}{c}1\,(n=0)\\ \frac{1}{\sqrt{2}}\,(n\ne 0)\end{array}$$

and

4 $${\phi }_{|n|}=\frac{{H}_{|n|}\,((x-{l}_{c}^{2}{k}_{y})/{l}_{c})}{\sqrt{{2}^{|n|}|n|!\sqrt{\pi }{l}_{c}}}\,{\rm{e}}{}^{[-\tfrac{1}{2}{(\tfrac{x-{l}_{c}^{2}{k}_{y}}{{l}_{c}})}^{2}]},$$

where ${l}_{c}=\sqrt{c\hslash /eB}$ is magnetic length and *H*_*n*_ (*x*) is the Hermite polynomial. The eigenenergy can be calculated as ${\varepsilon }_{n}={\rm{sgn}}\,(n)\,\hslash {\omega }_{c}\sqrt{|n|}$ with ${\omega }_{c}=\sqrt{2}{\upsilon }_{F}/{l}_{c}$. In comparison with LLs of a conventional 2*D* electron/hole system with a parabolic dispersion, LLs in graphene are unequally spaced and their transition energies are proportional to $\sqrt{B}$. Combining the eigenenergy of graphene system with the selected three energy levels in Fig. 2, we can simplify the system Hamiltonian without optical fields as

5 $${\hat{H}}_{0}=\hslash {\varepsilon }_{3}\,|3\rangle \langle 3|+\hslash {\varepsilon }_{2}\,|2\rangle \langle 2|+\hslash {\varepsilon }_{1}\,|1\rangle \langle 1|.$$

With including the light-matter interaction in the graphene system, the vector potential of the optical field ${\overrightarrow{A}}_{opt}=ic\overrightarrow{E}/\omega$ $(\overrightarrow{E}={\overrightarrow{E}}_{p}+{\overrightarrow{E}}_{TH})$ is employed into the vector potential of the magnetic field in the generalized momentum operator $\overrightarrow{\pi }$ in the Hamiltonian. The generated interaction Hamiltonian can be given by

6 $${\hat{H}}_{{\rm{int}}}={\upsilon }_{F}\overrightarrow{\sigma }\cdot \frac{e}{c}{\overrightarrow{A}}_{opt}.$$

The interaction Hamiltonian (6) does not include the momentum operator, and it is only determined by the Pauli matrix vector $\overrightarrow{\sigma }$ and proportional to vector potential ${\overrightarrow{A}}_{opt}$. The matrix element of the optical transition between LLs is given by

7 $$\langle m|{\hat{H}}_{{\rm{int}}}|n\rangle =\frac{i{\upsilon }_{F}}{\omega }\langle m|{\sigma }_{x}\hat{x}+{\sigma }_{y}\hat{y}|n\rangle \cdot \overrightarrow{E},$$

where |*m*〉, |*n*〉 are LLs with energy index *m* and *n*. The term $\langle m|{\sigma }_{x}\hat{x}+{\sigma }_{y}\hat{y}|n\rangle$ in Eq. (7) is

8 $$\sqrt{2}{C}_{m}{C}_{n}{(-i)}^{|m|+|n|-1}\{{\rm{sgn}}(m)\langle {\phi }_{|m|-1}|{\phi }_{|n|}\rangle \cdot {\hat{e}}_{LHS}+{\rm{sgn}}(n)\langle {\phi }_{|m|}|{\phi }_{|n|-1}\rangle \cdot {\hat{e}}_{RHS}\}.$$

Since *ϕ*_*n*_ are orthogonal, the above expression is nonzero only when |*m*| − 1 = |*n*| or |*m*| = |*n*| − 1. As a result, the selection rule for the allowed transitions turns out to be Δ|*n*| = ±1, where *n* is the energy quantum number.

Due to the special selection rules in present graphene, the selected transitions are dipole allowed between the appointed energy levels, i.e., Δ|*n*| = ±1 with *n* the energy quantum number. In case of Δ|*n*| = −1, the right-hand circularly (RHC) polarized photons could be homogeneously absorbed. Conversely, the left-hand circularly (LHC) polarized photons are simultaneously absorbed in case of Δ|*n*| = +1^3^. It should be noted that the carrier frequencies of optical transition between adjacent LLs turn out to be in the infrared or terahertz (THz) region for a magnetic field in the range of 0.01 − 10 T. The electric field vector of the system can be expressed as ${\overrightarrow{E}}_{p}={\overrightarrow{e}}_{-}{E}_{p}^{-}\,\exp \,(-i{\omega }_{p}t+i{\overrightarrow{k}}_{p}\cdot \overrightarrow{r})+c\mathrm{.}c\mathrm{.}$ and ${\overrightarrow{E}}_{TH}={\overrightarrow{e}}_{-}{E}_{TH}^{-}\,\exp \,(-i{\omega }_{TH}t+i{\overrightarrow{k}}_{TH}\cdot \overrightarrow{r})+c\mathrm{.}c\mathrm{.}$ with ${\overrightarrow{e}}_{-}({\overrightarrow{e}}_{+})$ the unit vector of the LHC (RHC) polarized basis. ${\overrightarrow{e}}_{-}({\overrightarrow{e}}_{+})$ can be noted as ${\overrightarrow{e}}_{-}=[\hat{x}-i\hat{y}]/\sqrt{2}({\overrightarrow{e}}_{+}=[\hat{x}+i\hat{y}]/\sqrt{2})$. In detail, the optical transition $|1\rangle \leftrightarrow |3\rangle \,(|2\rangle \leftrightarrow |3\rangle )$ is driven by the optical field of LHC polarized component ${E}_{p}^{-}({E}_{TH}^{-})$ with the carrier frequency *ω*_*p*_(*ω*_*TH*_).

**Figure 3:**
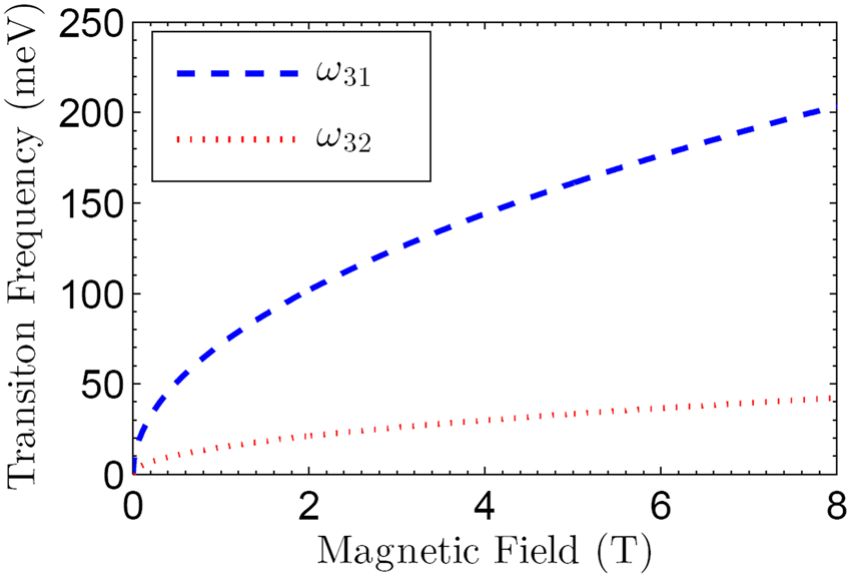
The transition frequencies in the three-level graphene system shown in Fig. 1(b). The transition frequency *ω*_31_ (*ω*_32_) indicates the splitting gap between levels |1〉 and |3〉 (|2〉 and |3〉).

The transition frequencies of relevant LLs can be estimated as ${\omega }_{31}=(\sqrt{2}+\mathrm{1)}{\omega }_{c}$ and ${\omega }_{32}=(\sqrt{2}-\mathrm{1)}{\omega }_{c}$. The Fig. 3 shows the transition frequency *ω*_31_ (*ω*_32_) between levels |1〉 and |3〉 (|2〉 and |3〉) as a function of the external magnetic field *B*. For the external magnetic field *B* up to 3 T, the frequency *ω*_*c*_ is on the order of ${\omega }_{c}\simeq {10}^{14}\,{s}^{-1}$. From Fig. 3, one can see that ${\omega }_{31}/2\pi \simeq 38.4$ THz (i.e., ${\omega }_{31}\simeq 2.41\times {10}^{14}\,{s}^{-1}$), which is located within the mid-infrared region. Accordingly, the transition frequency *ω*_32_/2*π* is about 6.59 THz (i.e., ${\omega }_{32}\simeq 4.14\times {10}^{13}\,{s}^{-1}$), which falls into the THz region.

By inserting the complete set of states {|3〉, |2〉, |1〉} in Eq. (6), we can obtain

9 $$\begin{array}{rcl}{\hat{H}}_{{\rm{int}}} & = & I\cdot {\upsilon }_{F}\overrightarrow{\sigma }\cdot \frac{e}{c}{\overrightarrow{A}}_{opt}\cdot I\\  & = & -{\mu }_{31}{E}_{p}^{-}{e}^{-i{\omega }_{p}t}|3\rangle \langle 1|-{\mu }_{32}{E}_{TH}^{-}{e}^{-i{\omega }_{TH}t}|3\rangle \langle 2|+h.c.\\  & = & -\hslash {{\rm{\Omega }}}_{p}{e}^{-i{\omega }_{p}t}|3\rangle \langle 1|-\hslash {{\rm{\Omega }}}_{TH}{e}^{-i{\omega }_{TH}t}|3\rangle \langle 2|+h.c.,\end{array}$$

where the complete set of states is $I={\sum }_{i}|i\rangle \langle i|$ (*i* = 1, 2, 3) and ${\overrightarrow{\mu }}_{mn}=\langle m|\overrightarrow{\mu }|n\rangle =e\cdot \langle m|\overrightarrow{r}|n\rangle =\frac{i\hslash e}{{\varepsilon }_{n}-{\varepsilon }_{m}}\langle m|{\upsilon }_{F}\overrightarrow{\sigma }|n\rangle$ denotes the dipole moments for the transition between states $|m\rangle \leftrightarrow |n\rangle$. The corresponding Rabi frequencies for the relevant laser-driven intersubband transitions are represented as ${{\rm{\Omega }}}_{p}=({\overrightarrow{\mu }}_{31}\cdot {\overrightarrow{e}}_{-}){E}_{p}^{-}/\hslash$ and ${{\rm{\Omega }}}_{TH}=({\overrightarrow{\mu }}_{32}\cdot {\overrightarrow{e}}_{-})\,{E}_{TH}^{-}/\hslash$.

Then we can obtain the total Hamiltonian of graphene system i.e., $\hat{H}={\hat{H}}_{0}+{\hat{H}}_{{\rm{int}}}$. In the interaction picture, with the rotating wave approximation and the electric dipole approximation, the total Hamiltonian of this system can be written as (assuming the state |1〉 as the zero potential reference and $\hslash =1$),

10 $${\hat{H}}_{int}^{I}={{\rm{\Delta }}}_{p}|3\rangle \langle 3|+({{\rm{\Delta }}}_{p}-{{\rm{\Delta }}}_{TH})|2\rangle \langle 2|-({{\rm{\Omega }}}_{p}|3\rangle \langle 1|+{{\rm{\Omega }}}_{TH}|3\rangle \langle 2|+h.c.),$$

where we define the frequency detunings ${{\rm{\Delta }}}_{p}=({\varepsilon }_{n=2}-{\varepsilon }_{n=-1})/\hslash -{\omega }_{p}$ and ${{\rm{\Delta }}}_{TH}=({\varepsilon }_{n=2}-{\varepsilon }_{n=1})/\hslash -{\omega }_{TH}$.

To give a full description of the dynamical evolution of our considered graphene system, we firstly adopt Liouville’s equation $\frac{\partial \hat{\rho }}{\partial t}=-\frac{i}{\hslash }\,[{\hat{H}}_{{\rm{int}}}^{I},\hat{\rho }]-\hat{R}(\hat{\rho })$. Here, $\hat{R}(\hat{\rho })=\frac{1}{2}\{\hat{{\rm{\Gamma }}},\hat{\rho }\}=\frac{1}{2}\{\hat{{\rm{\Gamma }}}\hat{\rho }+\hat{\rho }\hat{{\rm{\Gamma }}}\}$ indicates incoherent relaxation which stemming from disorder, interaction with phonon and carrier-carrier interactions. The density-matrix equations of motion for the present system can be written as follows:

11 $$\frac{\partial {\rho }_{11}}{\partial t}={\gamma }_{31}{\rho }_{33}+i{{\rm{\Omega }}}_{p}^{\ast }{\rho }_{31}-i{{\rm{\Omega }}}_{p}{\rho }_{13},$$

12 $$\frac{\partial {\rho }_{22}}{\partial t}={\gamma }_{32}{\rho }_{33}+i{{\rm{\Omega }}}_{TH}^{\ast }{\rho }_{32}-i{{\rm{\Omega }}}_{TH}{\rho }_{23},$$

13 $$\frac{\partial {\rho }_{33}}{\partial t}=-({\gamma }_{31}+{\gamma }_{32}){\rho }_{33}+i{{\rm{\Omega }}}_{p}{\rho }_{13}-i{{\rm{\Omega }}}_{p}^{\ast }{\rho }_{31}+i{{\rm{\Omega }}}_{TH}{\rho }_{23}-i{{\rm{\Omega }}}_{TH}^{\ast }{\rho }_{32},$$

14 $$\frac{\partial {\rho }_{31}}{\partial t}=-(\frac{{\gamma }_{31}+{\gamma }_{32}}{2}+i{{\rm{\Delta }}}_{p})\,{\rho }_{31}+i{{\rm{\Omega }}}_{p}({\rho }_{11}-{\rho }_{33})+i{{\rm{\Omega }}}_{TH}{\rho }_{21},$$

15 $$\frac{\partial {\rho }_{21}}{\partial t}=-(\frac{{\gamma }_{21}}{2}+i{{\rm{\Delta }}}_{p}-i{{\rm{\Delta }}}_{TH})\,{\rho }_{21}+i{{\rm{\Omega }}}_{TH}^{\ast }{\rho }_{31}-i{{\rm{\Omega }}}_{p}{\rho }_{23},$$

16 $$\frac{\partial {\rho }_{32}}{\partial t}=-(\frac{{\gamma }_{31}+{\gamma }_{21}}{2}+i{{\rm{\Delta }}}_{TH})\,{\rho }_{32}+i{{\rm{\Omega }}}_{TH}({\rho }_{22}-{\rho }_{33})+i{{\rm{\Omega }}}_{p}{\rho }_{12},$$

where the dephasing rate *γ*_*ij*_ (*i* = 1, 2, 3, $i\ne j$) is added phenomenologically in the above equations. For simplicity, we assume *γ*_31_ = *γ*_32_ = *γ* and ${\gamma }_{21}\ll \gamma$. For present system, the dephasing rate *γ* can be estimated numerically as *γ* = 3 × 10^13^ *s* ^−1^ according to refs 3, 33. We should note that the decay of the population of a certain LLs has been not included in the above equations. Compared to the dephasing rate, the population decay can be neglected (the lifetime of the carriers is in the picosecond range^5^). A comprehensive treatment of the decay rates would involve other broadening mechanism into the system. However, we have adopted the phenomenological approach of treating the decay just as done in the literatures^3, 9–12, 32^. A more fully treatment taking into account of these broadening mechanism has been investigated quite thoroughly by some authors (see, for example, refs 4–8).

In order to correctly describe the time-dependent dynamics of the infrared and THz pulse in the medium, equations of motion (11)–(16) must be simultaneously solved with Maxwell equation. As far as the propagation dynamics of two pulses is concerned, the following Maxwell wave equations in the slowly varying envelope approximation is required along the direction of $\hat{z}$:

17 $$\frac{\partial {E}_{p}^{-}(z,t)}{\partial z}+\frac{1}{c}\frac{\partial {E}_{p}^{-}(z,t)}{\partial t}=i\frac{{\omega }_{p}}{2c{\varepsilon }_{0}}{P}_{p}(z,t),$$

18 $$\frac{\partial {E}_{TH}^{-}(z,t)}{\partial z}+\frac{1}{c}\frac{\partial {E}_{TH}^{-}(z,t)}{\partial t}=i\frac{{\omega }_{TH}}{2c{\varepsilon }_{0}}{P}_{TH}(z,t),$$

where *c* and *ε*_0_ are the light speed and permittivity in free space, respectively. Without loss of generality, we consider one-dimensional propagation in this paper. *P*_*p*_(*z*, *t*) (*P*_*TH*_(*z*, *t*)) is the slowly oscillating term of the induced polarization in LLs transition between $|1\rangle (|2\rangle )\leftrightarrow |3\rangle$, and is determined by *P*_*p*_(*z*, *t*) = *Nμ*_31_ *ρ*_31_ (*P*_*TH*_(*z*, *t*) = *Nμ*_32_ *ρ*_32_). Note that the expressions of the polarization *P*_*p*_(*z*, *t*) (*P*_*TH*_(*z*, *t*)) contains a 2D electron density *N*. The electron density can be expressed as $N=2/(\pi {l}_{c}^{2})$ with the magnetic length *l*_*c*_. To convert them into the bulk polarization for comparison with other materials, we can divide it by the thickness of one monolayer as *N*_3*D*_ = *N*/Δ*z* (typically, Δ*z* = 0.34 nm). Of course there is no need to consider propagation of the pulses through a monolayer graphene. However, we have used the Maxwell wave equation to describe the propagation dynamics, which can keep our results general to make it applicable to a multiplayer graphene layer. In the present work, we will focus on the time-dependent control of the infrared and THz pulses for one monolayer thickness, i.e., *z* ~ Δ*z*.

## Results and Discussion

In this section, we would focus on the time-dependent coherent control of an infrared probe pulse and a THz switch pulse in the present system. We firstly assumed the infrared probe pulse is ${{\rm{\Omega }}}_{p}\mathrm{(0},t)$ at the entrance *z* = 0. In the limit of a weak probe signal, almost electrons will remain in the state |1〉 and hence we may assume that ${\rho }_{11}\mathrm{(0)}\approx 1$, and ${\rho }_{22,33}\mathrm{(0)}\approx 0$. Under this assumption, we arrive at the linearized results for time-dependent dynamics of the infrared probe field at the output terminal *z* = Δ*z* as,

19 $${{\rm{\Omega }}}_{p}(z,t)={{\rm{\Omega }}}_{p}\mathrm{(0},t){e}^{\alpha {\rm{\Delta }}z({\gamma }_{31}+{\gamma }_{32}){\rm{Im}}({\rho }_{31}/{{\rm{\Omega }}}_{p})},$$

where $\alpha =\frac{N{\omega }_{p}{|{\mu }_{31}|}^{2}}{4\hslash {\varepsilon }_{0}c({\gamma }_{31}+{\gamma }_{32})}$ is the propagation constant. Thus we can obtain the normalized absorption coefficient of the infrared probe pulse as $1-{{\rm{\Omega }}}_{p}(z,t)/{{\rm{\Omega }}}_{p}\mathrm{(0},t)$. We can directly examine the transient absorption property of the infrared probe pulse by numerically integrating Eqs (11)–(16) with a certain initial condition. With the initial conditions ${\rho }_{11}\mathrm{(0)}\approx 1$, ${\rho }_{\mathrm{22,33}}\mathrm{(0)}\approx 0$, and ${\rho }_{ij}\mathrm{(0)}\approx 0$ for $i\ne j$ (*i*, *j* = 1, 2, 3), we solve the time-dependent Eqs (11)–(16) by a standard fourth-order Runge-Kutta method.

**Figure 4:**
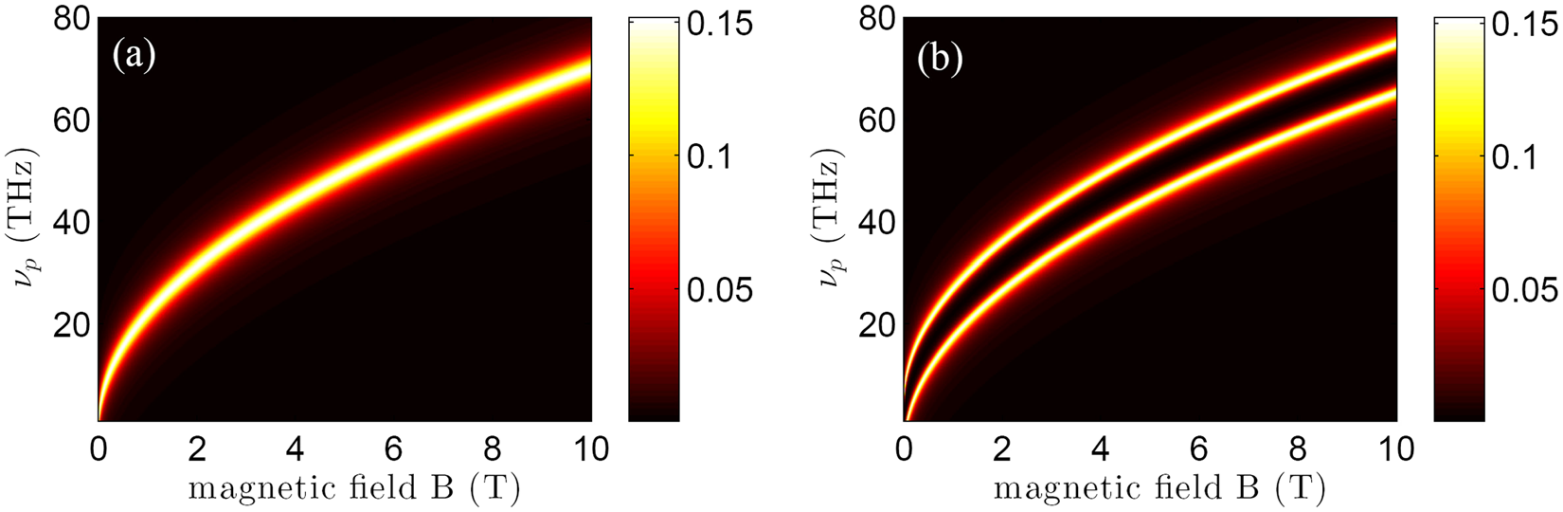
Contour maps of the absorption $1-{{\rm{\Omega }}}_{p}(z,t)/{{\rm{\Omega }}}_{p}\mathrm{(0},t)$ as a function of the probe frequency ${\nu }_{p}={\omega }_{p}\mathrm{/2}\pi$ and the magnetic field intensity *B*. (**a**) In absence of the THz switch pulse (i.e., ${{\rm{\Omega }}}_{TH}=0$); (**b**) In presence of the THz switch pulse (i.e., ${{\rm{\Omega }}}_{TH}=\gamma$). Other parameters are given as *γ*_31_ = *γ*_32_ = *γ*, *γ* = 3 × 10^13^ *s* ^−1^, *γ*_21_ = 0.05*γ*, ${{\rm{\Omega }}}_{p}=0.05\gamma$, Δ_*p*_ = 0, Δ_*TH*_ = 0, and Δ*z* = 0.34 nm.

For a directly insight into the modulation of the THz switch pulse on the absorption of the infrared probe pulse with different magnetic field intensity *B*, we now numerically simulate the absorption spectra versus the frequency of the probe pulse and the magnetic field intensity for two different cases, i.e., ${{\rm{\Omega }}}_{TH}=0$ and ${{\rm{\Omega }}}_{TH}=\gamma$. In addition, the frequency detunings of infrared probe and THz switch pulses, as well as the intensity of probe pulse are chosen as Δ_*p*_ = 0, Δ_*TH*_ = 0, and ${{\rm{\Omega }}}_{p}=0.05{\gamma }_{3}$. According to above practical parameter sets, we plot the absorption coefficients of the probe pulse $1-{{\rm{\Omega }}}_{p}(z,t)/{{\rm{\Omega }}}_{p}\mathrm{(0},t)$ as a function of the probe frequency ${\nu }_{p}={\omega }_{p}/2\pi$ and the magnetic field intensity *B* without and with including THz switch pulse, as shown in Fig. 4. It can be seen from Fig. 4 that the absorption spectra can be modulated by the secondary THz switch pulse. Figure 4(a), corresponds to the case of $|{{\rm{\Omega }}}_{TH}|=0$, shows that the only high absorption line appears in the center of the probe absorbtion spectra. With the increasing of magnetic field intensity *B*, there is a shift about the center frequency of the probe absorption spectra peak in mid-infrared region. However, when the secondary THz switch pulse is switched on, Fig. 4(b) shows that the original high absorption line becomes a obvious transparency window between two high absorption lines. In other words, the secondary THz switch pulse can be regarded as a switching for controlling the absorption of the infrared probe pulse. These interesting phenomena results from the destructive interference induced by the secondary THz switch pulse. The mechanism of this destructive interference is similar as the one in EIT medium^13–15^. However, interestingly, the parameters of the splitting gap of quantized LLs in graphene can be engineered to give a desired transmission by utilizing applied magnetic field in design.

**Figure 5:**
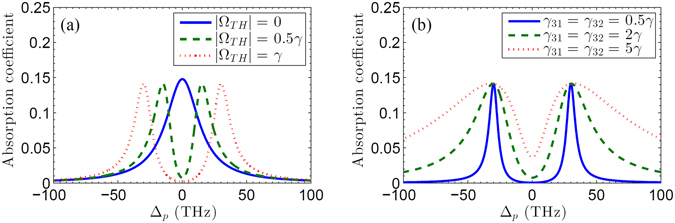
(**a**) The absorption coefficient $1-{{\rm{\Omega }}}_{p}(z,t)/{{\rm{\Omega }}}_{p}(0,t)$ as a function of the frequency frequency Δ_*p*_ (THz) with *γ*_31_ = *γ*_32_ = *γ* for different values of ${{\rm{\Omega }}}_{TH}$, i.e., ${{\rm{\Omega }}}_{TH}=0$ (solid line), ${{\rm{\Omega }}}_{TH}=0.5\gamma$ (dashed line), and ${{\rm{\Omega }}}_{TH}=1\gamma$ (dotted line), respectively. (**b**) The absorption coefficient $1-{{\rm{\Omega }}}_{p}(z,t)/{{\rm{\Omega }}}_{p}\mathrm{(0},t)$ as a function of the frequency frequency Δ_*p*_ (THz) with ${{\rm{\Omega }}}_{TH}=\gamma$ for different values of *γ*_31,32_, i.e., *γ*_31_ = *γ*_32_ = 0.5*γ* (solid line), *γ*_31_ = *γ*_32_ = 2*γ* (dashed line), and *γ*_31_ = *γ*_32_ = 5*γ* (dotted line), respectively. Other parameters are given as *B* = 3 T, *γ* = 3 × 10^13^ *s* ^−1^, *γ*_21_ = 0.05*γ*, ${{\rm{\Omega }}}_{p}=0.05\gamma$, Δ_*TH*_ = 0, and Δ*z* = 0.34 nm.

In order to provide a clear picture for the destructive interference induced by the THz switch pulse, for a fixed external magnetic field *B* = 3 T, we plot in Fig. 5(a) the absorption coefficient of the infrared probe pulse as the function of the frequency detuning Δ_*p*_ for different values of ${{\rm{\Omega }}}_{TH}$, i.e., ${{\rm{\Omega }}}_{TH}=0$, ${{\rm{\Omega }}}_{TH}=0.5\gamma$, and ${{\rm{\Omega }}}_{TH}=\gamma$, respectively. As can be seen from Fig. 5(a), the absorption curves depend sensitively on the value of ${{\rm{\Omega }}}_{TH}$. In the absence of the THz switch pulse, i.e., ${{\rm{\Omega }}}_{TH}=0$, a obvious absorption peak around the probing resonance position (Δ_*p*_ = 0) can be observed in the absorption curve. When the THz switch beam is on (${{\rm{\Omega }}}_{TH}=0.5\gamma$), a narrow transparency window occurs around probing resonance position. As the value of ${{\rm{\Omega }}}_{TH}$ increases from 0.5*γ* to *γ*, the transparency window becomes wide correspondingly. Note that we have chosen a certain value of the dephasing rate. As a matter of fact, the dephasing is determined by many complex factors^8^, such as radiative broadening, Coulomb-induced broadening, broadening due to the scattering with optical phonons, and impurity-induced broadening. We plot in Fig. 5(b) absorption profiles of the infrared probe pulse for different values of the dephasing rates compared them with the ones shown in Fig. 5(a). One can find that the transparency window becomes wide if the values of dephasing rates (*γ*_31_ = *γ*_32_) decrease from *γ* to 0.5*γ*. And also, Fig. 5(b) shows that the increasing of the dephasing rates can lead to an intensive disturbance for the transparency window. In other words, a high-quality graphene with small dephasing rate may provide a practical help to build the destructive interference and control the absorption property of the infrared probe pulse even if the THz switch pulse is on.

It should be borne in mind that the above destructive interference mechanism can be understood according to the classical dressed-state theory^34^. When the transition $|2\rangle \leftrightarrow |3\rangle$ is driven by the THz switch pulse, the state |3〉 will split into two dressed states |*a*〉 and |*b*〉 with *λ*_*a*_ and *λ*_*b*_ the corresponding energy eigenvalues, respectively. With the rotating-wave approximation and the electric dipole approximation, the Hamiltonian of the transition $|2\rangle \leftrightarrow |3\rangle$ with the THz switch pulse can be given as ($\hslash =1$) ${\hat{H}}_{int}^{I}={{\rm{\Delta }}}_{TH}|3\rangle \langle 3|-({{\rm{\Omega }}}_{TH}|3\rangle \langle 2|+{{\rm{\Omega }}}_{TH}^{\ast }|2\rangle \langle 3|)$ in the interaction picture. By solving eigenvalues equation, we can obtain the two energy eigenvalues of the two dressed states as

20 $${\lambda }_{a}=\frac{1}{2}({{\rm{\Delta }}}_{TH}+\sqrt{{{\rm{\Delta }}}_{TH}^{2}+4{{\rm{\Omega }}}_{TH}^{2}}),$$

21 $${\lambda }_{b}=\frac{1}{2}({{\rm{\Delta }}}_{TH}-\sqrt{{{\rm{\Delta }}}_{TH}^{2}+4{{\rm{\Omega }}}_{TH}^{2}}).$$

Accordingly, the dressed states shown in the inset of Fig. 2 can be given by

22 $$|a\rangle =-\cos \,{\theta }_{1}|3\rangle +\,\sin \,{\theta }_{1}|2\rangle ,$$

23 $$|b\rangle =\,\sin \,{\theta }_{1}|3\rangle +\,\cos \,{\theta }_{1}|2\rangle $$

with

24 $$\sin \,{\theta }_{1}=\frac{{\lambda }_{a}}{\sqrt{{\lambda }_{a}^{2}+{{\rm{\Omega }}}_{TH}^{2}}}=\frac{{{\rm{\Omega }}}_{TH}}{\sqrt{{\lambda }_{b}^{2}+{{\rm{\Omega }}}_{TH}^{2}}},$$

25 $$\cos \,{\theta }_{1}=-\frac{{\lambda }_{b}}{\sqrt{{\lambda }_{b}^{2}+{{\rm{\Omega }}}_{TH}^{2}}}=\frac{{{\rm{\Omega }}}_{TH}}{\sqrt{{\lambda }_{a}^{2}+{{\rm{\Omega }}}_{TH}^{2}}}.$$

Considering the resonance condition (i.e., Δ_*TH*_ = 0), the energy eigenvalues of Eqs (20) and (21) can be rewritten as ${\lambda }_{a}=+{{\rm{\Omega }}}_{TH}$ and ${\lambda }_{b}=-{{\rm{\Omega }}}_{TH}$, respectively. Correspondingly, the dressed states of Eqs (22) and (23) can be rewritten as ${\lambda }_{a}=(|2\rangle +|3\rangle )/\sqrt{2}$ and ${\lambda }_{a}=(|2\rangle -|3\rangle )/\sqrt{2}$, respectively. That is, the splitting of state |3〉 is evenly spaced when the THz switch field is resonant coupled to the transition $|2\rangle \leftrightarrow |3\rangle$, which holds the similar mechanism as the ac-stark splitting. The probe dipole matrix elements corresponding to the transition $|1\rangle \leftrightarrow |3\rangle$ in this dressed picture can be represented as

26 $${d}_{a}=\langle a|P|1\rangle =-{\mu }_{31}/\sqrt{2},$$

27 $${d}_{b}=\langle b|P|1\rangle ={\mu }_{31}/\sqrt{2}$$

with *P* = *μ*_31_|3〉 〈1|. Obviously, depending on the frequency detuning Δ_*p*_ of the infrared probe pulse and energy eigenvalues *λ*_*a*_ and *λ*_*b*_, the absorption of the infrared probe pulse can be modulated by the secondary THz switch pulse. When the frequency detuning of the infrared probe pulse is tuned at ${{\rm{\Delta }}}_{p}={\lambda }_{a}={{\rm{\Omega }}}_{TH}$ or ${{\rm{\Delta }}}_{p}={\lambda }_{b}=-{{\rm{\Omega }}}_{TH}$, two resonant excitations happen through the channels in the dressed state basis $|1\rangle \leftrightarrow |a\rangle$ and $|1\rangle \leftrightarrow |b\rangle$. Consequently, the two absorption peaks occur at the position ${{\rm{\Delta }}}_{p}=\pm {{\rm{\Omega }}}_{TH}$ and a transparency window can be observed around the position Δ_*p*_ = 0, just as illustrated in Fig. 5.

As illustrated in Figs 4 and 5, we have shown that the destructive interference can be constructed in the present graphene system under Landau quantization. We also demonstrated that absorption of the infrared probe pulse can be controlled by the secondary THz switch pulse and the applied magnetic field intensity. In order to further study the time-dependent coherent control of an infrared probe pulse and a THz switch pulse via this destructive interference effect in the present system. We assume two pulses’ space-time-dependent Rabi frequencies as

28 $${{\rm{\Omega }}}_{p}(z,t)={{\rm{\Omega }}}_{p}^{0}f(z,t),$$

29 $${{\rm{\Omega }}}_{TH}(z,t)={{\rm{\Omega }}}_{TH}^{0}g(z,t)$$

are here written in terms of the peak Rabi frequencies ${{\rm{\Omega }}}_{p}^{0}$ and ${{\rm{\Omega }}}_{TH}^{0}$ and the dimensionless infrared probe and THz switch pulse envelopes *f*(*z*, *t*) and *g*(*z*, *t*) with durations *τ*_*p*_ and *τ*_*TH*_, respectively. In the local (retarded) frame where *ξ* = *z* and *τ* = *t* − *z*/*c*, according to $\partial /\partial z=\partial /\partial \xi -\mathrm{1/}c\partial /\partial \tau$ and $\partial /\partial t=\partial /\partial \tau$, the equations of motion for the density matrix elements *ρ*_*ij*_(*ξ*, *τ*) and the wave equations for the normalized infrared probe and THz switch pulses *f*(*ξ*, *τ*) and *g*(*ξ*, *τ*) across the graphene system can be rewritten as

30 $$\frac{\partial {\rho }_{11}}{\partial \tau }={\gamma }_{31}{\rho }_{33}+i{{\rm{\Omega }}}_{p}^{0}{f}^{\ast }(\xi ,\tau ){\rho }_{31}-i{{\rm{\Omega }}}_{p}^{0}f(\xi ,\tau ){\rho }_{13},$$

31 $$\frac{\partial {\rho }_{22}}{\partial \tau }={\gamma }_{32}{\rho }_{33}+i{{\rm{\Omega }}}_{TH}^{0}g(\xi ,\tau ){\rho }_{32}-i{{\rm{\Omega }}}_{TH}^{0}{g}^{\ast }(\xi ,\tau ){\rho }_{23},$$

32 $$\begin{array}{rcl}\frac{\partial {\rho }_{33}}{\partial \tau } & = & -({\gamma }_{31}+{\gamma }_{32}){\rho }_{33}+i{{\rm{\Omega }}}_{p}^{0}f(\xi ,\tau ){\rho }_{13}-i{{\rm{\Omega }}}_{p}^{0}{f}^{\ast }(\xi ,\tau ){\rho }_{31}\\  &  & +i{{\rm{\Omega }}}_{TH}^{0}g(\xi ,\tau ){\rho }_{23}-i{{\rm{\Omega }}}_{TH}^{0}{g}^{\ast }(\xi ,\tau ){\rho }_{32},\end{array}$$

33 $$\frac{\partial {\rho }_{31}}{\partial \tau }=-(\frac{{\gamma }_{31}+{\gamma }_{32}}{2}+i{{\rm{\Delta }}}_{p}){\rho }_{31}+i{{\rm{\Omega }}}_{p}^{0}f(\xi ,\tau )({\rho }_{11}-{\rho }_{33})+i{{\rm{\Omega }}}_{TH}^{0}g(\xi ,\tau ){\rho }_{21},$$

34 $$\frac{\partial {\rho }_{21}}{\partial \tau }=-(\frac{{\gamma }_{21}}{2}+i{{\rm{\Delta }}}_{p}-i{{\rm{\Delta }}}_{TH}){\rho }_{21}+i{\Omega }_{TH}^{0}{g}^{\ast }(\xi ,\tau ){\rho }_{31}-i{\Omega }_{p}^{0}f(\xi ,\tau ){\rho }_{23},$$

35 $$\frac{\partial {\rho }_{32}}{\partial \tau }=-(\frac{{\gamma }_{31}+{\gamma }_{21}}{2}+i{{\rm{\Delta }}}_{TH}){\rho }_{32}+i{{\rm{\Omega }}}_{TH}^{0}g(\xi ,\tau )({\rho }_{22}-{\rho }_{33})+i{{\rm{\Omega }}}_{p}^{0}f(\xi ,\tau ){\rho }_{12},$$

36 $$\frac{\partial f(\xi ,\tau )}{\partial \alpha \xi }=i\frac{{\gamma }_{31}+{\gamma }_{32}}{{{\rm{\Omega }}}_{p}^{0}}{\rho }_{31}(\xi ,\tau ),$$

37 $$\frac{\partial g(\xi ,\tau )}{\partial \beta \xi }=i\frac{{\gamma }_{31}+{\gamma }_{21}}{{{\rm{\Omega }}}_{TH}^{0}}{\rho }_{32}(\xi ,\tau ),$$

where $\alpha =\frac{N{\omega }_{p}{|{\mu }_{31}|}^{2}}{4\hslash {\varepsilon }_{0}c({\gamma }_{31}+{\gamma }_{32})}$ and $\beta =\frac{N{\omega }_{TH}{|{\mu }_{32}|}^{2}}{4\hslash {\varepsilon }_{0}c({\gamma }_{31}+{\gamma }_{21})}$ denote the propagation coefficients of the infrared probe and THz switch pulses, respectively. We further assume that the infrared probe and THz switch pulses are assumed as Gaussian-type pulses at *ξ* = 0, i.e., $f(\xi =0,\tau )=\exp \,[-\mathrm{2(}\,\mathrm{ln}\,\mathrm{2)(}\tau -{\mathrm{160)}}^{2}/{\tau }_{p}^{2}]$ and $g(\xi =0,\tau )=\exp \,[-\mathrm{2(}\,\mathrm{ln}\,2){(\tau -\mathrm{150)}}^{2}/{\tau }_{s}^{2}]$. In the following, we solve the coupled Bloch-Maxwell equations (30)–(37) by using the iterative predictor-corrector finite-difference time-domain method with the initial condition that all the electrons start in the ground state |1〉.

**Figure 6:**
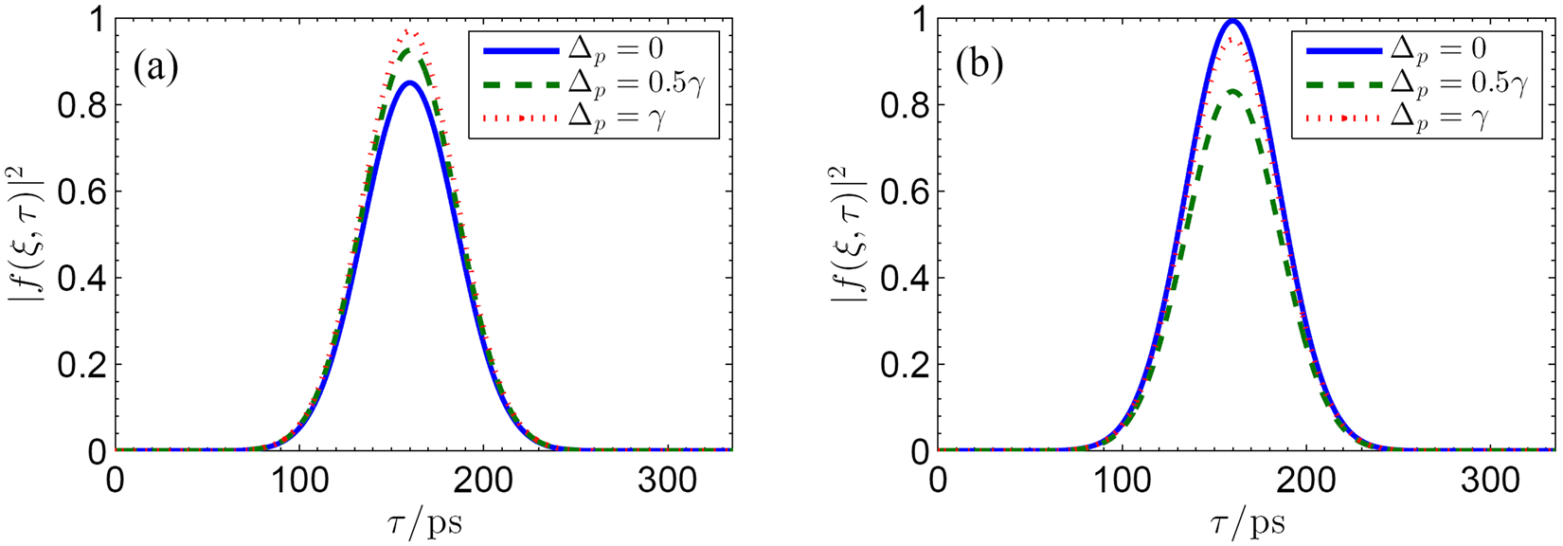
Snapshots of the temporal evolution of the infrared probe pulse at output terminal (*ξ* ~ Δ*z*) for different cases, i.e., (**a**) ${{\rm{\Omega }}}_{TH}^{0}=0$, (**b**) ${{\rm{\Omega }}}_{TH}^{0}=0.5\gamma$. The solid line, dashed line, and dotted line correspond to Δ_*p*_ = 0, 0.5*γ* and *γ*, respectively. Other parameters are given as *B* = 3 T, *γ*_31_ = *γ*_32_ = *γ*, *γ* = 3 × 10^13^ *s* ^−1^, *γ*_21_ = 0.05*γ*, ${{\rm{\Omega }}}_{p}^{0}=0.05\gamma$, Δ_*TH*_ = 0, *τ*_*p*_ = 60 *ps*, *τ*_*s*_ = 100 *ps*, $f(\xi =0,\tau )=\exp [-\mathrm{2(}\,\mathrm{ln}\,\mathrm{2)(}\tau -{\mathrm{160)}}^{2}/{\tau }_{p}^{2}]$ and $g(\xi =0,\tau )=\exp \,[-\mathrm{2(}\,\mathrm{ln}\,\mathrm{2)(}\tau -{\mathrm{150)}}^{2}/{\tau }_{s}^{2}]$.

We start by reporting in Fig. 6 the temporal evolutions of the normalized probe pulse |*f*(*ξ*, *τ*)|^2^ at output terminal (*ξ* ~ Δ*z*) for different frequency detuning of the infrared probe pulse, i.e., Δ_*p*_ = 0, 0.5*γ* and *γ*, respectively. This is done for two different cases, i.e., without (${{\rm{\Omega }}}_{TH}^{0}=0$) and with (${{\rm{\Omega }}}_{TH}^{0}=\gamma$) including THz switch pulse. It can be easily seen that the time evolution of the infrared probe pulse depends prominently on the THz switch pulse. In the absence of the secondary THz switch pulse (i.e., ${{\rm{\Omega }}}_{TH}^{0}=0$), the obvious absorption can be observed from Fig. 6(a) when the transition $|1\rangle \leftrightarrow |3\rangle$ is coupled resonantly by the infrared probe pulse (Δ_*p*_ = 0). If the frequency detuning Δ_*p*_ is tuned to be off-resonance from the corresponding transition, the absorption of the Gaussian-type probe pulse will be suppressed. In presence of the secondary THz switch pulse with ${{\rm{\Omega }}}_{TH}^{0}=\gamma$, one can find from Fig. 6(b) that the Gaussian-type probe pulse exhibits perfect transmission without absorption when Δ_*p*_ = 0 due to the existence of the destructive interference, which agrees with the results shown in Fig. 5(a). These results are also similar as the EIT in atomic systems, where destructive interference between two optical absorption paths of a probe pulse can be controlled by a cw pump field in Λ-type atoms^13–15^. However, different from the EIT effect, we extend the destructive interference to the pulsed regime, where the destructive interference between the optical transitions (|1〉 $\leftrightarrow$ |3〉 and |2〉 $\leftrightarrow$ |3〉) in the present graphene was achieved by controlling the THz switch pulse.

**Figure 7:**
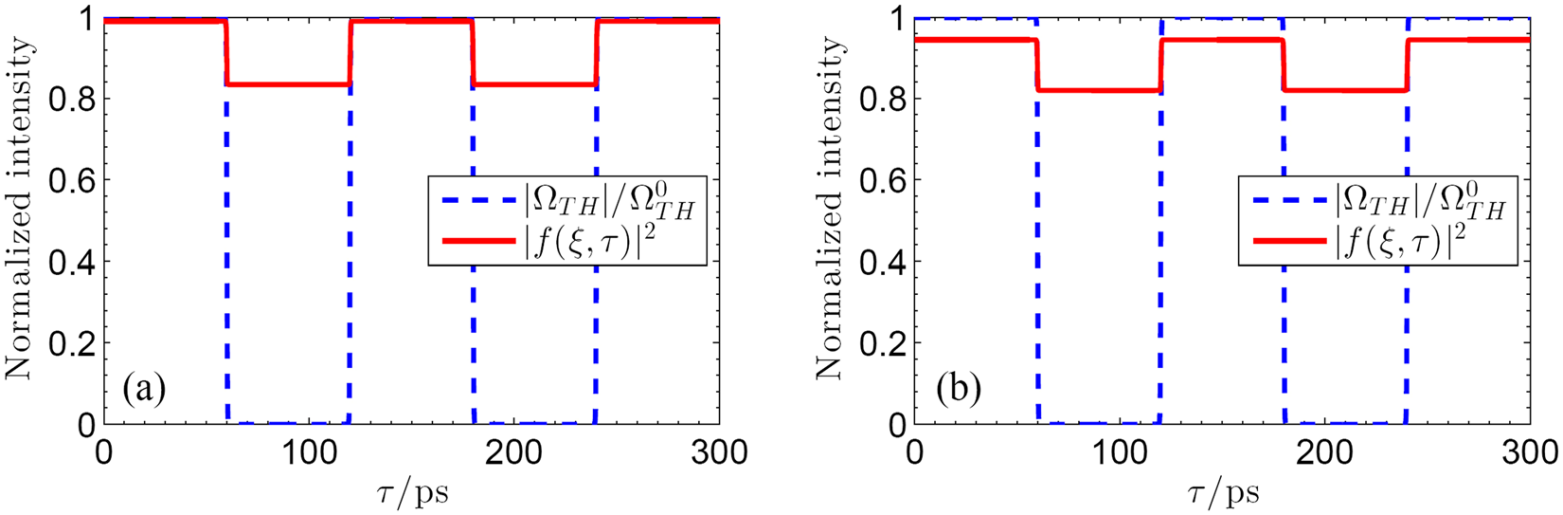
Time evolution of the infrared probe pulse (solid line) at output terminal *ξ* ~ Δ*z* driven by a THz switch pulse with wave form ${{\rm{\Omega }}}_{TH}={{\rm{\Omega }}}_{TH}^{0}[1-0.5\,\tanh \,\mathrm{[4(}\tau -\mathrm{60)]}+0.5\,\tanh \,\mathrm{[4(}\tau -\mathrm{120)]}$ − 0.5 tanh $\mathrm{[4(}\tau -\mathrm{180)]}+0.5\,\tanh \,\mathrm{[4(}\tau -\mathrm{240)]]}$ for different frequency detuning Δ_*p*_: (**a**) Δ_*p*_ = 0; (**b**) Δ_*p*_ = 0.5*γ*. Other parameters are *f*(*ξ* = 0, *τ*) = 1, *B* = 3 T, *γ*_31_ = *γ*_32_ = *γ*, *γ* = 3 × 10^13^ *s* ^−1^, *γ*_21_ = 0.05*γ*, ${{\rm{\Omega }}}_{p}^{0}=0.05\gamma$, Δ_*TH*_ = 0, and ${{\rm{\Omega }}}_{TH}^{0}=\gamma$, respectively.

As discussed above, the absorption of the infrared probe pulse can be modulated by the secondary THz pulse. Thus we can devise optical switching via choosing an appropriate wave form of THz switch pulse. In the following, we will show a representative result for the implement of optical switching by choosing the switch THz pulse as a square wave train. Without the loss of generality, the wave form of a THz switch pulse can be given by ${{\rm{\Omega }}}_{TH}={{\rm{\Omega }}}_{TH}^{0}[1-0.5\,\tanh \,\mathrm{[4(}\tau -\mathrm{60)]}+0.5\,\tanh \,\mathrm{[4(}\tau -\mathrm{120)]}$ − $0.5\,\tanh \,\mathrm{[4(}\tau -\mathrm{180)]}+0.5\,\tanh \,\mathrm{[4(}\tau -240)]]$. By numerically solving Eqs (30)–(37), we can finally examine the time evolution of the infrared probe pulse. Figure 7 shows that the time evolution of the normalized pulse envelopes of infrared probe pulse in presence of the above THz switch pulse for different frequency detuning Δ_*p*_. One can find that there is a steplike transition between absorption and transparency of the infrared probe pulse. Therefore, the absorption and transparency of the infrared pulse can be switched on and off by devising the secondary THz pulse. And this optical switching provides an convenient way to controlling time-dependent evolution of infrared probe pulse in graphene with Landau quantization. In addition, Fig. 7 also shows that the steplike transition between absorption and transparency of the infrared probe pulse can be controlled by the frequency detuning Δ_*p*_. As mentioned in the above section, the carrier frequencies of optical transition between adjacent LLs are determined by the external magnetic field *B*. As a result, by suitably varying the magnetic field *B*, we can realize magnetic-optical modulation for such a infrared probe pulse.

**Figure 8:**
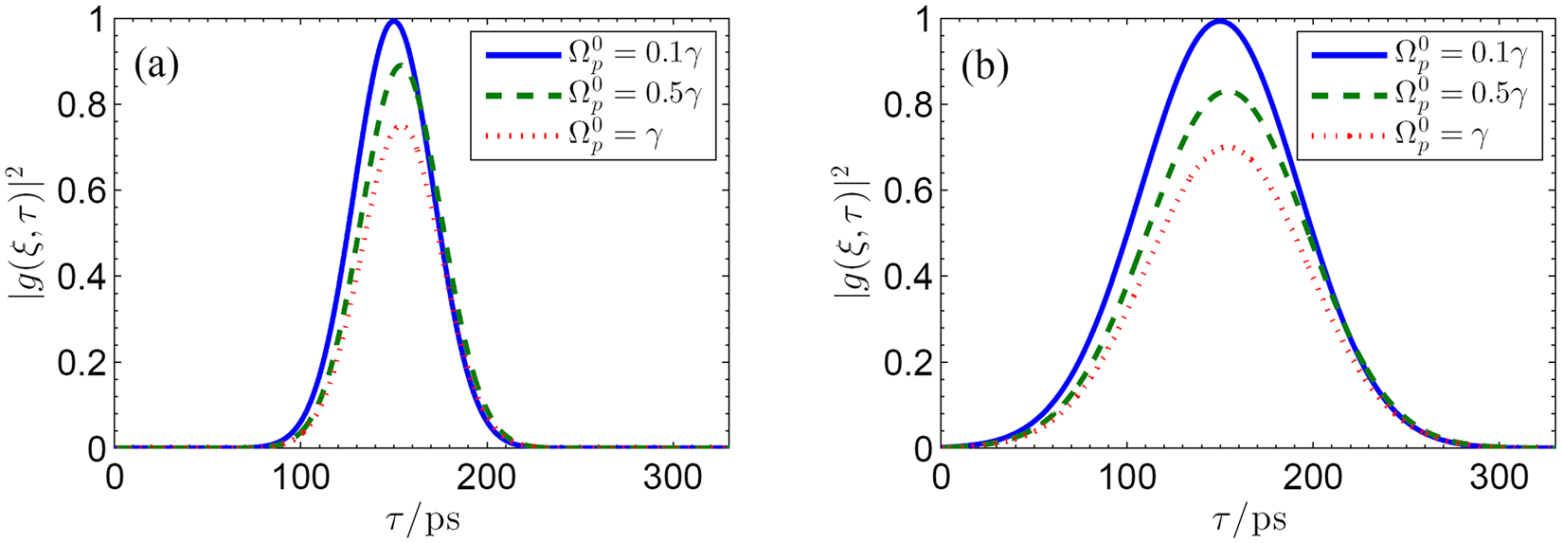
Snapshots of the temporal evolution of the switch THz pulse output terminal (*ξ* ~ Δ*z*) for different cases, i.e., (**a**) *τ*_*p*_ = *τ*_*s*_ = 100 ps; (**b**) *τ*_*p*_ = *τ*_*s*_ = 50 ps. The solid line, dashed line, and dotted line correspond to ${{\rm{\Omega }}}_{p}^{0}=0.1\gamma$, ${{\rm{\Omega }}}_{p}^{0}=0.5\gamma$, and ${{\rm{\Omega }}}_{p}^{0}=\gamma$, respectively. Other parameters are given as *B* = 3 T, *γ*_31_ = *γ*_32_ = *γ*, *γ* = 3 × 10^13^ *s* ^−1^, *γ*_21_ = 0.05*γ*, ${{\rm{\Omega }}}_{TH}^{0}=0.005\gamma$, Δ_*p*_ = Δ_*TH*_ = 0, $f(\xi =0,\tau )=\exp \,[-\mathrm{2(}\,\mathrm{ln}\,\mathrm{2)(}\tau -{\mathrm{160)}}^{2}/{\tau }_{p}^{2}]$ and $g(\xi =0,\tau )=\exp \,[-\mathrm{2(}\,\mathrm{ln}\,\mathrm{2)(}\,\tau -{\mathrm{150)}}^{2}/{\tau }_{s}^{2}]$.

**Figure 9:**
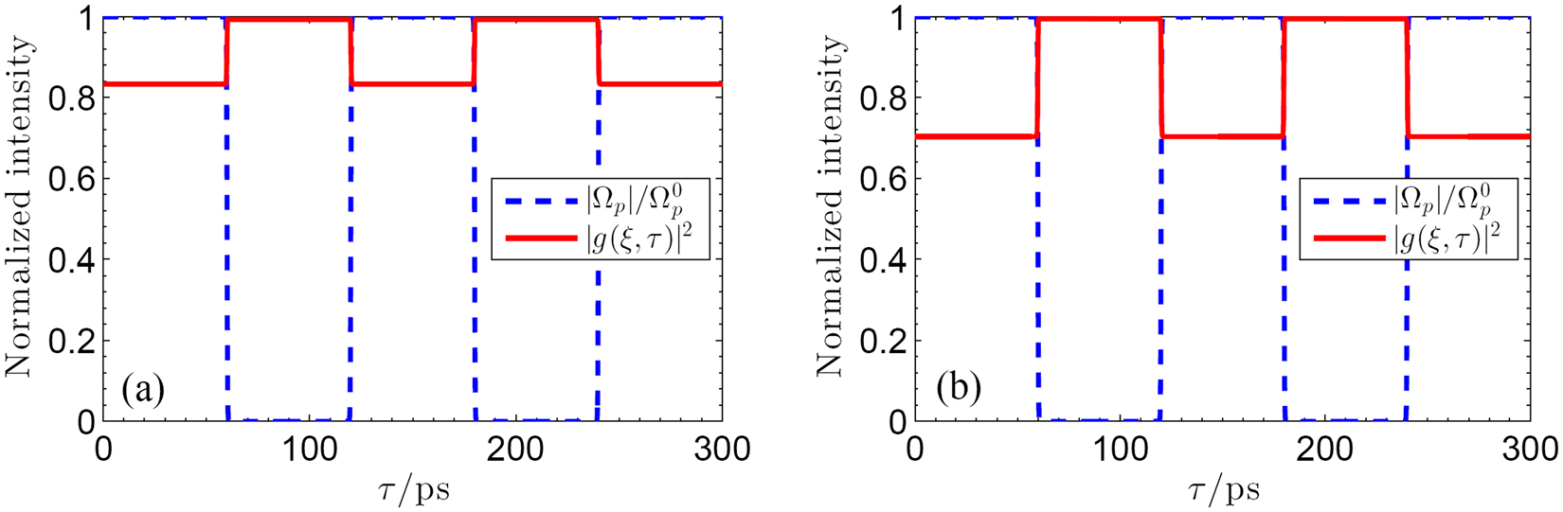
Time evolution of the THz pulse (solid line) at the penetration depth *ξ* = 10/*β* in the sample driven by an infrared probe pulse with wave form ${{\rm{\Omega }}}_{p}={{\rm{\Omega }}}_{p}^{0}\,[1-0.5\,\tanh \,\mathrm{[4(}\tau -\mathrm{60)]}+0.5\,\tanh \,\mathrm{[4(}\tau -\mathrm{120)]}$ − 0.5 tanh $\mathrm{[4(}\tau -\mathrm{180)]}+0.5\,\tanh \,\mathrm{[4(}\tau -\mathrm{240)]]}$ for different ${{\rm{\Omega }}}_{p}^{0}$: (**a**) ${{\rm{\Omega }}}_{p}^{0}=0.5\gamma$; (**b**) ${{\rm{\Omega }}}_{p}^{0}=\gamma$. Other parameters are *g*(*ξ* = 0, *τ*) = 1, *B* = 3 T, *γ*_31_ = *γ*_32_ = *γ*, *γ* = 3 × 10^13^ *s* ^−1^, *γ*_21_ = 0.05*γ*, ${{\rm{\Omega }}}_{TH}^{0}=0.005\gamma$, and Δ_*p*_ = Δ_*TH*_ = 0, respectively.

Up to now, we have investigated the temporal evolution of infrared probe pulse which depends prominently on the THz switch pulse. Interestingly, the reverse case can also be realized precisely where a THz switch pulse controlled by a secondary probe pulse is rendered either opaque or transparent to the present graphene system. As an example, we consider in Fig. 8 the reverse case where a infrared probe controls the temporal evolutions of the normalized THz switch pulse |*g*(*ξ*, *τ*)|^2^ for different widths of two pulses (i.e., *τ*_*p*_ = *τ*_*s*_ = 100 ps and *τ*_*p*_ = *τ*_*s*_ = 50 ps) with varying the probe pulse intensity ${{\rm{\Omega }}}_{p}^{0}$ (i.e., ${{\rm{\Omega }}}_{p}^{0}=0.1\gamma$, ${{\rm{\Omega }}}_{p}^{0}=0.5\gamma$, and ${{\rm{\Omega }}}_{p}^{0}=\gamma$, respectively). The switch THz pulse can be severely attenuated by the infrared probe pulse, which makes the switch THz pulse behave as a signal and the infrared probe pules as a control beam. However, it is worth noting that the physics mechanism differs from the case shown in Fig. 6. Here, when the infrared probe pulse is weak, the THz switch pulse propagates with completely transparency due to the lack of populations in states |2〉 and |3〉. In contrast, when the infrared probe pulse is strong, the THz switch pulse is attenuated predominately owing to the induced absorption process whereby one photon from each of two beams is absorbed, exciting an electron from state |1〉 to state |3〉. In addition, the time evolution of the THz switch pulse is very sensitive to the pulse widths. Direct comparison in Fig. 8(a,b) implies that the narrower spectrum makes the attenuation or absorption of THz switch pulse more pronounced, which can be explained using the time-dependent perturbation theory^34^. As done in Fig. 7, we show in Fig. 9 that the time evolution of the normalized pulse envelopes of THz switch pulse by choosing the wave form of a probe pulse as ${{\rm{\Omega }}}_{p}={{\rm{\Omega }}}_{p}^{0}[1-0.5\,\tanh \,\mathrm{[4(}\tau -\mathrm{60)]}+0.5\,\tanh \,\mathrm{[4(}\tau -\mathrm{120)]}$ − $0.5\,\tanh \,\mathrm{[4(}\tau -\mathrm{180)]}+0.5\,\tanh \,\mathrm{[4(}\tau -\mathrm{240)]]}$. One can find that there is also a steplike transition between absorption and transparency of the THz pulse. In other words, the absorption and transparency of the THz pulse can be controlled by devising the secondary infrared pulse, which provides an convenient way to controlling time-dependent evolution of THz pulse in graphene with Landau quantization.

## Conclusion

In conclusion, we have investigated in detail the dynamics control of two coherent pulses, an infrared probe and a terahertz (THz) switch pulses, in monolayer graphene where the destructive interference may take place and be easily tuned. We analyze the physics mechanism of this destructive interference indcued by one of the two pulses in graphene under Landau quantization in a time-dependent way via the Bloch-Maxwell formalism. Based on this analysis, we find that the graphene system can be completely transparent to the infrared probe pulse when such an interference induced by the switch pulse take places. In the absence of the switch pulse, the infrared probe pulse can pass through the monolayer graphene with partial transparency. In the presence of the switch pulse, the absorption can be modulated from optimizing the destructive interference by varying the intensity of switch pulse and frequency detuning of the infrared probe pulse. In addition, we provide a clear physics insight of optimizing destructive interference by using the classical dressed-state theory. Conversely, the present graphene system may be rendered either absorbing or transparent to the THz switch pulse. By choosing the appropriate wave form of the pulses, we have demonstrated that both infrared probe and THz switch pulses can exhibit the steplike transitions between absorption and transparency. The present results illustrated the potential to utilize destructive interference to realize the time-dependent control for either signal or control with distinct wave-lengths which may turn out to be useful in designing quantum interference-based solid-state devices for optical communications.
